# Upregulation of SK3 and IK1 Channels Contributes to the Enhanced Endothelial Calcium Signaling and the Preserved Coronary Relaxation in Obese Zucker Rats

**DOI:** 10.1371/journal.pone.0109432

**Published:** 2014-10-10

**Authors:** Belén Climent, Laura Moreno, Pilar Martínez, Cristina Contreras, Ana Sánchez, Francisco Pérez-Vizcaíno, Albino García-Sacristán, Luis Rivera, Dolores Prieto

**Affiliations:** 1 Departamento de Fisiología, Facultad de Farmacia, Universidad Complutense, Madrid, Spain; 2 Departamento de Farmacología, Facultad de Medicina, Universidad Complutense, Madrid, Spain; 3 Departamento de Anatomía y Anatomía Patológica Comparadas, Facultad de Veterinaria, Universidad Complutense, Madrid, Spain; University of Hull, United Kingdom

## Abstract

**Background and Aims:**

Endothelial small- and intermediate-conductance K_Ca_ channels, SK3 and IK1, are key mediators in the endothelium-derived hyperpolarization and relaxation of vascular smooth muscle and also in the modulation of endothelial Ca^2+^ signaling and nitric oxide (NO) release. Obesity is associated with endothelial dysfunction and impaired relaxation, although how obesity influences endothelial SK3/IK1 function is unclear. Therefore we assessed whether the role of these channels in the coronary circulation is altered in obese animals.

**Methods and Results:**

In coronary arteries mounted in microvascular myographs, selective blockade of SK3/IK1 channels unmasked an increased contribution of these channels to the ACh- and to the exogenous NO- induced relaxations in arteries of Obese Zucker Rats (OZR) compared to Lean Zucker Rats (LZR). Relaxant responses induced by the SK3/IK1 channel activator NS309 were enhanced in OZR and NO- endothelium-dependent in LZR, whereas an additional endothelium-independent relaxant component was found in OZR. Fura2-AM fluorescence revealed a larger ACh-induced intracellular Ca^2+^ mobilization in the endothelium of coronary arteries from OZR, which was inhibited by blockade of SK3/IK1 channels in both LZR and OZR. Western blot analysis showed an increased expression of SK3/IK1 channels in coronary arteries of OZR and immunohistochemistry suggested that it takes place predominantly in the endothelial layer.

**Conclusions:**

Obesity may induce activation of adaptive vascular mechanisms to preserve the dilator function in coronary arteries. Increased function and expression of SK3/IK1 channels by influencing endothelial Ca^2+^ dynamics might contribute to the unaltered endothelium-dependent coronary relaxation in the early stages of obesity.

## Introduction

Endothelial calcium-activated K (K_Ca_) channels, including small conductance (SK3 or K_Ca_2.3) and intermediate conductance (IK1 or K_Ca_3.1) isoforms, are important effectors modulating arterial tone, since their opening is a starting point in the so-called non-chemical endothelial-derived hyperpolarization (EDH). This response causes vascular smooth muscle (VSM) relaxations resistant to nitric oxide (NO) synthases (NOS) and cyclooxygenases inhibitors [1], [2] and implies electrotonical coupling between endothelial and VSM cells which leads to VSM hyperpolarization and relaxation [1], [3]. The EDH-mediated response is initiated with the increase in endothelial intracellular Ca^2+^ concentration ([Ca^2+^]_i_ which activates SK3 and IK1 channels and causes endothelial cell hyperpolarization [2], [4]. Besides the role of SK3 and IK1 channels in the non-chemical EDH response, it is now well established that activation of these channels increases the driving force for Ca^2+^ entry into endothelial cells [5]–[7]. This implies that SK3/IK1 channel-mediated hyperpolarization of endothelial cell itself can modulate activation of endothelial NOS and thus NO release and relaxation [8]–[11].

Obesity is associated with cardiovascular and metabolic disorders such as insulin resistance, impaired glucose tolerance, hypertension, and dyslipidemia, jointly referred to as metabolic syndrome. Each of these disorders is an independent predictor of cardiovascular events thus, obese patients have increased prevalence of cardiovascular co-morbidities e.g. type 2 diabetes, hyperlipidemia, hypertension, heart disease, and stroke as well as inflammation [12]. Obesity is associated with endothelial dysfunction and impaired relaxation [12], [13] which has mainly been ascribed to the diminished bioavailability of endothelium-derived NO as a consequence of increased reactive oxygen species formation and of the abnormal profile of proinflammatory cytokines release from the inflamed adipose tissue [14]–[17]. In contrast, EDH-mediated relaxation appears to persist or even compensate for the loss of NO-mediated relaxation under obesity conditions [18]–[21].

Concerning coronary endothelial function in obesity, clinical and experimental studies have shown preserved basal coronary blood flow [22] and unaltered, attenuated, or even augmented vasodilator responses to endothelial agonists in coronary arterioles from humans [23] and experimental models of obesity [24]–[26]. This has led to the suggestion that coronary arteries initially adapt to match the higher metabolic demand in obesity by preserving their vasodilator function and they are somehow “resistant” to the early endothelial dysfunction that occurs in other vascular beds [27].

Endothelium-dependent relaxation in large coronary arteries seems to be mainly due to NO release [28] while the contribution of EDH-mediated responses is of larger importance in small coronary arteries [29]. Regarding the role of K_Ca_ channels in the endothelium-dependent relaxations of coronary arteries in obesity both impaired [30] and preserved function [31] have been reported for the large conductance K_Ca_ (BK_Ca_) channels, while the function of endothelial SK3/IK1 channels remains largely unexplored. In this regard, we have previously described preserved endothelial relaxations mediated by NO in coronary arteries of an animal model of genetic obesity and insulin resistance [26], [32]. In order to better understand the mechanisms underlying this preserved coronary endothelial response, the present study was designed to assess whether SK3 and IK1 channels contribute to the endothelial NO-mediated relaxation in coronary arteries, and if so, to determine whether these channels may be involved in the signaling pathway for this preservation.

## Methods

### 1.1. Ethics Statement

This study was conducted in compliance with the European Directive for the Protection of Animals Used for Scientific Purposes (2010/63/EU). All animal care and experimental protocols were approved by ethical committee of Complutense University of Madrid (Spain).

### 1.2. Animal model

Male Obese Zucker Rats (fa/fa, OZR) and their counterpart, Lean Zucker Rats (fa/-, LZR) were provided from Charles River Laboratories (Barcelona, Spain). Animals were anesthetized with sodium pentobarbital (50 mg/kg, i.p.) and euthanized by decapitation and exsanguination. The depth of anesthesia was evaluated by pinching the animal's paw with forceps and all efforts were made to minimize suffering. The heart was quickly removed and placed in cold (4°C) physiological saline solution (PSS) of the following composition (mM): 119 NaCl, 4.7 KCl, 1.18 KH_2_PO_4_, 1.17 MgSO_4_, 1.5 CaCl_2_, 24.9 NaHCO_3_, 0.027 EDTA, and 11 glucose; bubbled with a mixture of 95% O_2_ and 5% CO_2_, resulting in a pH 7.4.

### 1.3. Isolation and mounting of small coronary arteries

Intramyocardial second order branches of the left anterior descending coronary artery were carefully dissected as previously described [32], [33]. Arterial segments from OZR and LZR were mounted in microvascular myographs (DMT, Denmark) and equilibrated at 37°C. For each individual artery, the internal circumference L100 corresponding to a transmural pressure of 100 mmHg for a relaxed vessel *in situ* was calculated. The arteries were set to an internal circumference L_1_ equal to 0.9 times L100, at which tension development is maximal [32].

### 1.4. Experimental procedures for the functional assays

At the beginning of each experiment, coronary arteries were challenged twice with 124 mM K^+^ (KPSS) equivalent to PSS except that NaCl was exchanged for KCl on an equimolar basis. Endothelium-dependent relaxations were evaluated in serotonin (5-HT)-precontracted coronary arteries from LZR and OZR by constructing concentration-dependent response curves to acetylcholine (ACh, 10 nM-30 µM) in the absence and presence of the NOS inhibitor L-NG nitro arginine (L-NOARG, 100 µM), the selective SK3 channel blocker apamin (0.5 µM), the selective IK1 channel blocker 1-[(2-chlorophenyl)diphenylmethyl]-1H-pyrazole (TRAM34), (0.4 µM), and a combination of apamin plus TRAM34. Since ACh-induced relaxations in coronary arteries of both LZR and OZR are mostly mediated by NO [26], the effect of SK3 and IK1 channel blockers was also assessed on the relaxations to the NO donor S-nitroso-N-Acetylpenicillamine (SNAP) (10 nM-30 µM), in order to determine whether SK3/IK1 channels might be involved in NO relaxant actions in VSM. Since 5-HT-induced contraction was enhanced in arteries from OZR [26], 5-HT was applied in a range of concentrations (1-3 µM) in order to match the levels of precontraction in LZR and OZR. Arteries were incubated with the inhibitors in the myogragh chamber for 30 minutes.

In order to assess whether SK3/IK1 activation is involved in endothelial NO release in coronary arteries, the effect of the SK3/IK1 channel opener 6,7-dichloro-1H-indole-2,3-dione 3-oxime (NS309) was tested in 5-HT-precontracted coronary arteries in the presence and in the absence of endothelium, and under conditions of NOS blockade with L-NOARG. The compound NS309 exhibits a slight selectivity for IK1 over SK3 channels and has no effect on BK_Ca_ channels [34]. NS309 has been reported to block smooth muscle L-type voltage-dependent Ca^2+^ channels at high concentrations [34], [35] and therefore doses of this compound higher than 3 µM were not used in the present study. The endothelium was mechanically removed by guiding a human hair in the vessel lumen and the absence of functional endothelium in these arteries was confirmed by the lack of relaxation elicited by ACh (10 µM).

### 1.5. In situ measurements of endothelial [Ca^2+^] _i_

Measurements of endothelial [Ca^2+^]_i_ were performed in intact arterial segments by Fura-2 acetoxymethyl ester (Fura2-AM) fluorescence as previously described [36]. In order to allow access to the endothelial layer, coronary arterial segments were mounted in a wire myograph with a U shape cut out on the wall facing down. The myograph was mounted on an inverted microscope (Axiovert S100 TV) equipped for dual excitation wavelength microfluorimetry (Deltascan, Photon Technology International). For the measurements of endothelial [Ca^2+^]_i_, coronary arteries were loaded in the dark in PSS containing 4 µM Fura2-AM, 40 µg/ml pluronic acid and 0.05% Cremophor EL for 30 minutes at 37°C in order to selectively load the endothelial cell layer [36]. After loading, arteries were washed three times with fresh PSS every 10 minutes during 30 minutes and then illuminated with alternating 340 and 380 nm light using a monochromator based system and the intensity of the emitted fluorescence was collected at a wavelength of 510 nm using a photomultiplier and monitored. At the end of each experiment, Ca^2+^-insensitive signals were determined after quenching with Mn^2+^ and the values obtained were subtracted from those recorded during the experiment. The ratio (R) of fluorescence at 340 and 380 nm (F_340_/F_380_) corrected for autofluorescence was taken as a measure of [Ca^2+^]_i_. Ratiometric measurements compensate for the fading in the F340 and F380 fluorescent signals over time. The relative change in the fluorescent signal upon agonist stimulation was calculated as the increase in the ratio baseline prior to addition of the agonist to the level reached after 5-8 min of stimulation (ΔF_340_/_380_). Ratio min values were 0.4±0.09 and 0.62±0.08 and ratio max values were 2.17±0.97 and 2.95±0.51 in LZR and OZR respectively (n = 5 and n = 6).The effect of 3 µM ACh and the potent transient receptor potential vanilloid 4 (TRPV4) channel agonist, (N-((1S)-1-{[4-((2S)-2-{[(2,4-Dichlorophenyl)sulfonyl]amino}-3-hydroxypropanoyl)-1-piperazinyl]carbonyl}-3-methylbutyl)-1-benzothiophene-2-carboxamide (GSK1016790a) [37] was evaluated in endothelial cells from intact coronary arteries, in the absence and in the presence of apamin plus TRAM34.

Measurements of *in situ* endothelial [Ca^2+^]_i_ in intact arteries were performed in the continuous presence of nifedipine (0.5 µM) to quench the coronary smooth muscle Ca^2+^ signal, largely-dependent on L-type Ca^2+^ channels [33]. In endothelium-denuded segments of the coronary artery there was no measurable FURA-2 signal (n = 3).

### 1.6. Western blotting

The whole left descending coronary artery was dissected from the myocardium of LZR (n = 7) and OZR (n = 5), snap frozen in liquid nitrogen and homogenized under reducing condition in the presence of DTT and proteases inhibitors. Protein content was determined by Bio-Rad DC Protein Assay Kit (Bio-Rad, CA, USA) and equal amounts of proteins (20 µg) were loaded and subjected to electrophoresis on a SDS-PAGE (7.5%) followed by transference to a PVDF membrane (Bio-Rad). Protein expression was quantified using primary antibodies anti-SK3 (Alomone, Israel, 1∶200 dilution), anti-IK1 (Alomone, Israel, 1∶450 dilution), or anti-β-actin as a loading control (Sigma-Aldrich, Spain, 1∶10000 dilution) and horseradish peroxidase conjugated secondary goat anti-mouse and anti-rabbit antibodies (Santa Cruz Biotech, CA, USA, 1∶10000 dilution). Proteins were detected using ECL-Plus Western blotting reagents (Amersham, CT, USA) and analyzed using Quantity One (BioRad) [32]. Relative intensity for each protein was determined by comparison with the intensity of β-actin staining on blots that were stripped and then reprobed with β-actin primary antibody.

### 1.7. Immunohistochemistry

Tissue samples from the heart containing the left descending coronary artery were immersion-fixed in 4% paraformaldehyde in 0.1 M sodium phosphate-buffer (PB), cryoprotected in 30% sucrose in PB, embedded and frozen in OCT compound (Tissue-Tek, Sakura Finetek, Europe BV) and stored at −80°C. Cryostat sections 5–10 µm thick were obtained and preincubated in 10% normal goat serum in PB containing 0.3% Triton-X-100 for 2–3 h. Then, sections were incubated with either a rabbit anti-SK3 channel antibody (Alomone Labs, Israel) diluted at 1∶100 or a rabbit anti-IK1 channel antibody (Alomone Labs, Israel) diluted at 1∶100 for 48 h. Endothelial layer was visualized by coimmunostaining for eNOS with a mouse anti-eNOS antibody (Chemicon International Inc, 1∶500 dilution). Sections were then washed and reacted with the second antibodies for 2 h at room temperature. Secondary antibodies used were Alexa Fluor 594 (red) goat-anti-rabbit (Invitrogen, Life Technologies S.A., Spain, 1∶200 dilution) and Alexa Fluor 488 (green) goat-antimouse (Invitrogen, Great Britain, 1∶200 dilution). No immunoreactivity could be detected in sections incubated in the absence of the primary antisera. Preadsorption with IK1 and SK3 channels showed no cross-reactivity for the antibodies.

### 1.8. Drugs

The sources of the compounds used were as follows: acetylcholine, apamin, GSK101679a and serotonin were obtained from Sigma Aldrich (Spain); NS309, SNAP and TRAM34 were obtained from Tocris (Great Britain); Fura2-AM and ionomycin were obtained from Invitrogen (Great Britain). Stock solutions of GSK101679a, NS309, SNAP and TRAM34 were dissolved in DMSO and further diluted in distilled water. The final volume of DMSO never exceeded 0.01% in organ baths and did not affect smooth muscle tone in control experiments. All the other drugs were dissolved in distilled water.

### 1.9. Statistical analysis and data presentation

In the functional experiments, mechanical responses of the arteries were measured as force and expressed as active wall tension. Results are expressed as either absolute values (mN/mm of tension or units of R [F_340_/F_380_]) or as a percent of constrictor response to 5-HT in each artery. Data are expressed as means ± SEM (n  =  number of arteries, 1–2 from each animal in the vasoreactivity experiments, 1 from each animal in the measurements of endothelial [Ca^2+^]_i_, and 1 from each animal in the western blotting and immunohistochemical studies). pEC_50_ is −log EC_50_, EC_50_ was the concentration of agonist giving half maximal response (E_max_). The differences between means were analyzed using one way ANOVA followed by a Bonferroni post-test or paired or unpaired Student's t test when appropriate. Probability levels smaller than 5% were considered significant. All calculations were made using a standard software package (Prism 5.0, GraphPad, CA, USA).

## Results

### 2.1. General parameters

At 17–18 weeks of age, OZR displayed a significant increase in body weight compared with LZR and exhibited mild hyperglycemia and hyperinsulinemia along with elevated total cholesterol and triglyceride levels (Table S1).

The normalized internal lumen diameters, l_1_, of coronary arteries in the OZR group (332±13 µm, n = 39, 1–2 arterial segments per animal) were not significantly different from those in the LZR group (309±13 µm, n = 43, 1–2 arterial segments per animal). The standard contractions evoked by a high K^+^ solution were also similar in the LZR (0.55±0.07 Nm^−1^, n = 39) and in the OZR group (0.63±0.06 Nm^−1^, n = 43).

### 2.2. Contribution of SK3 and IK1 channels to ACh-induced relaxation is increased in intramyocardial arteries from OZR

**Figure 1:**
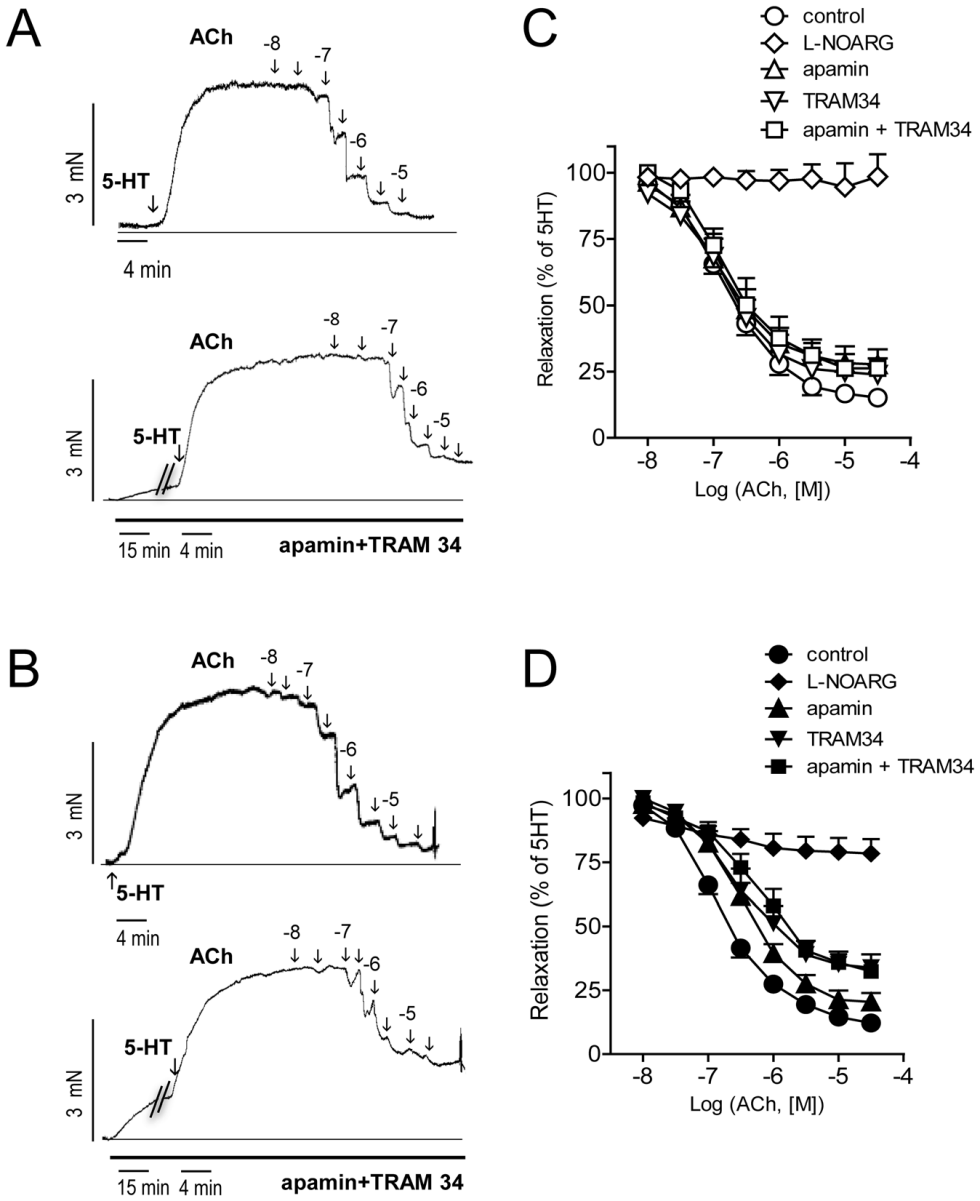
Increased contribution of SK3 and IK1 channels to the ACh-induced relaxation in coronary arteries from OZR. (A, B) Isometric tension recordings showing the effects of increasing concentrations of acetylcholine (ACh) on coronary arteries precontracted with 5-HT (1–3 µM) and effect of treatment with the SK3 channel inhibitor apamin (0.5 µM) plus the IK1 channel inhibitor TRAM34 (0.4 µM) in LZR (A) and OZR (B). (C–D) Effects of apamin, TRAM34, apamin plus TRAM34 and L-NOARG (100 µM) on the average concentration-dependent curves for the relaxation to ACh in coronary arteries from LZR (C) and OZR (D). Results are expressed as a percentage of the contraction induced by 5-HT. Points represent mean ± SEM of n = 6–9 arteries (1–2 arteries per animal).

ACh induced relaxations of similar magnitude in coronary arteries from LZR and OZR, as previously reported [25], [26], [32]. NOS inhibition with L-NOARG increased basal tone in both LZR and OZR arteries, and ACh-induced relaxations were abolished in LZR and reduced by about 80% in OZR (Fig. 1, Table 1), suggesting that NO mediates these relaxations in both healthy and obese animals. In this regard, we aimed to explore whether SK3/IK1 channels contribute to the ACh-induced NO-mediated relaxation of coronary arteries, thus the effects of selective inhibitors of SK3 (apamin) and IK1 (TRAM34) channels were assessed. In LZR arteries, incubation with apamin slightly decreased sensitivity and maximal response to ACh, while treatment with TRAM34 or apamin plus TRAM34 affected maximal relaxant response (Fig. 1 C, Table 1). However, apamin and TRAM 34, either alone or in combination, reduced to a larger extent both sensitivity and maximal response to ACh in arteries from obese animals (Fig. 1 D, Table 1). Furthermore, treatment with TRAM34 either alone or in combination with apamin increased basal tone in arteries of OZR (Table 1) indicating an enhanced basal activity of IK1 channels in obese animals.

**Table 1:** Effect of selective inhibitors of NOS (L-NOARG), SK3 (apamin) and IK1 channels (TRAM 34), and combined inhibition of SK3/IK1 channels (A+T) on the ACh and SNAP-induced vasodilation in coronary arteries from LZR and OZR.

ACh								
		*LZR*				*OZR*		
	p*EC_50_*	*E_max_*	*ΔBT*	*n*	p*EC_50_*	*E_max_*	*ΔBT*	*n*
**control**	7.01±0.19	91±3	-	7	6.9±0.15	90±5	-	6
**L-NOARG**	-	2±8^ c^	25±14	7	-	22±6^c^	31±13	6
**control**	6.8±0.16	87±6	-	7	6.8±0.13	93±2	-	8
**apamin**	7.0±0.18^ a^	73±5^ a^	9±3	7	6.42±0.10^a^	80±4^b^	14±5	8
**control**	7.0±0.2	90±2	-	6	6.9±0.19	86±3	-	9
**TRAM34**	6.9±0.13	73±6^ a^	9±6	6	6.43±0.16^a^	65±5^c^	25±6^†^	9
**control**	6.9±0.22	89±6	-	9	6.9±0.25	90±4	-	6
**A+T**	6.8±0.19	74±3^ b^	10±2	9	6.1±0.14^b^	68±1^c^	35±8^#^	6

SNAP								
		*LZR*				*OZR*		
	*p*EC_50_	*E_max_*	*ΔBT*	*n*	p*EC_50_*	*E_max_*	*ΔBT*	*n*
**control**	6.73±0.07	89±3	-	8	6.77±0.17	91±3	-	7
**apamin**	6.62±0.11	74±3^c^	8±2	8	6.76±0.14	75±3^a^	9±1	7
**control**	6.82±0.19	87±5	-	7	6.82±0.16	85±6	-	6
**TRAM34**	6.82±0.22	71±5^a^	10±5	7	-	34±4^a^	32±9^†^	6

Values represent mean ± S.E.M. of the number *n* of individual arteries. pEC_50_ is –logEC_50_. E_max_ is maximal relaxation expressed as percentage of 5-HT-induced precontraction. *ΔBT;* is the increase in basal tension expressed as percentage of KPSS-induced contraction. Significant differences from controls were analyzed using paired t test; ^a^ *P <*0.05; ^b^ *P <*0.01; ^c^ *P <*0.001 *vs* control; or one way ANOVA followed by a Bonferroni test for across-group comparisons; ^#^ *P <*0.05 *vs A*+T-treated LZR; ^†^ *P <*0.05 *vs* TRAM34-treated LZR.

**Figure 2:**
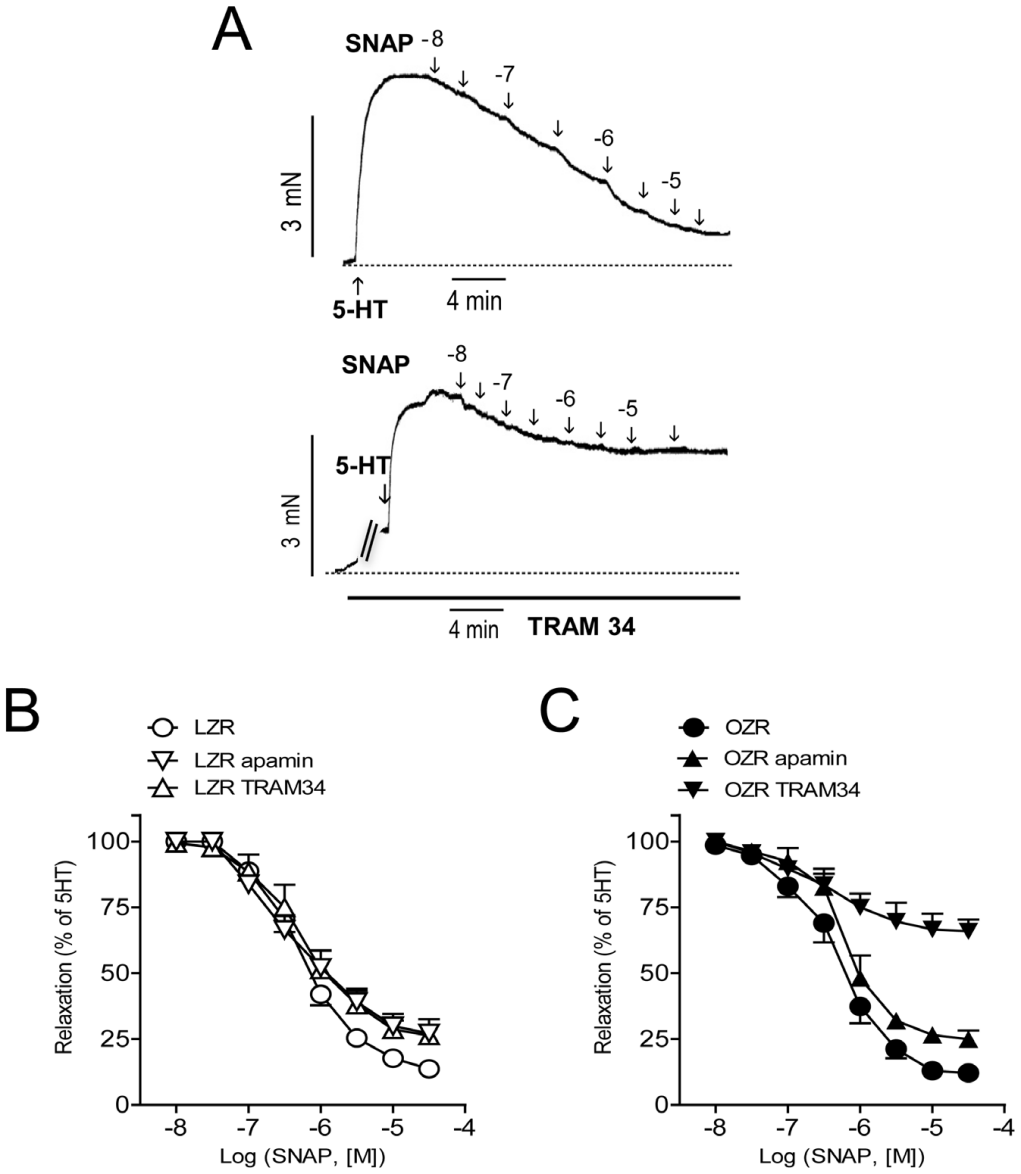
Increased contribution of IK1 channels to the SNAP-induced relaxations in coronary arteries from OZR. (A) Isometric tension recordings showing the effects of increasing concentrations of SNAP on coronary arteries precontracted with 5-HT (1–3 µM) and the effect of treatment with TRAM34 (0.4 µM) in OZR. (B, C) Effect of apamin (0.5 µM) and TRAM34 on the average concentration-dependent curves for the relaxation to SNAP in coronary arteries of LZR (B) and OZR (C). Results are expressed as a percentage of the contraction induced by 5-HT. Points represent mean ± SEM of n = 6–8 arteries (1–2 arteries per animal).

A possible contribution of SK3 and IK1 channels to the NO-mediated coronary relaxation was assessed by testing the effect of SK3/IK1 channel blockers on relaxations to the NO donor SNAP. Apamin modestly decreased SNAP-elicited relaxations in arteries from both LZR and OZR, whereas TRAM34 induced a marked decrease of NO relaxant responses in arteries from OZR (Fig 2, Table 1).

### 2.3. Role of SK3 and IK1 channels activation in the NO-induced coronary relaxation

**Figure 3:**
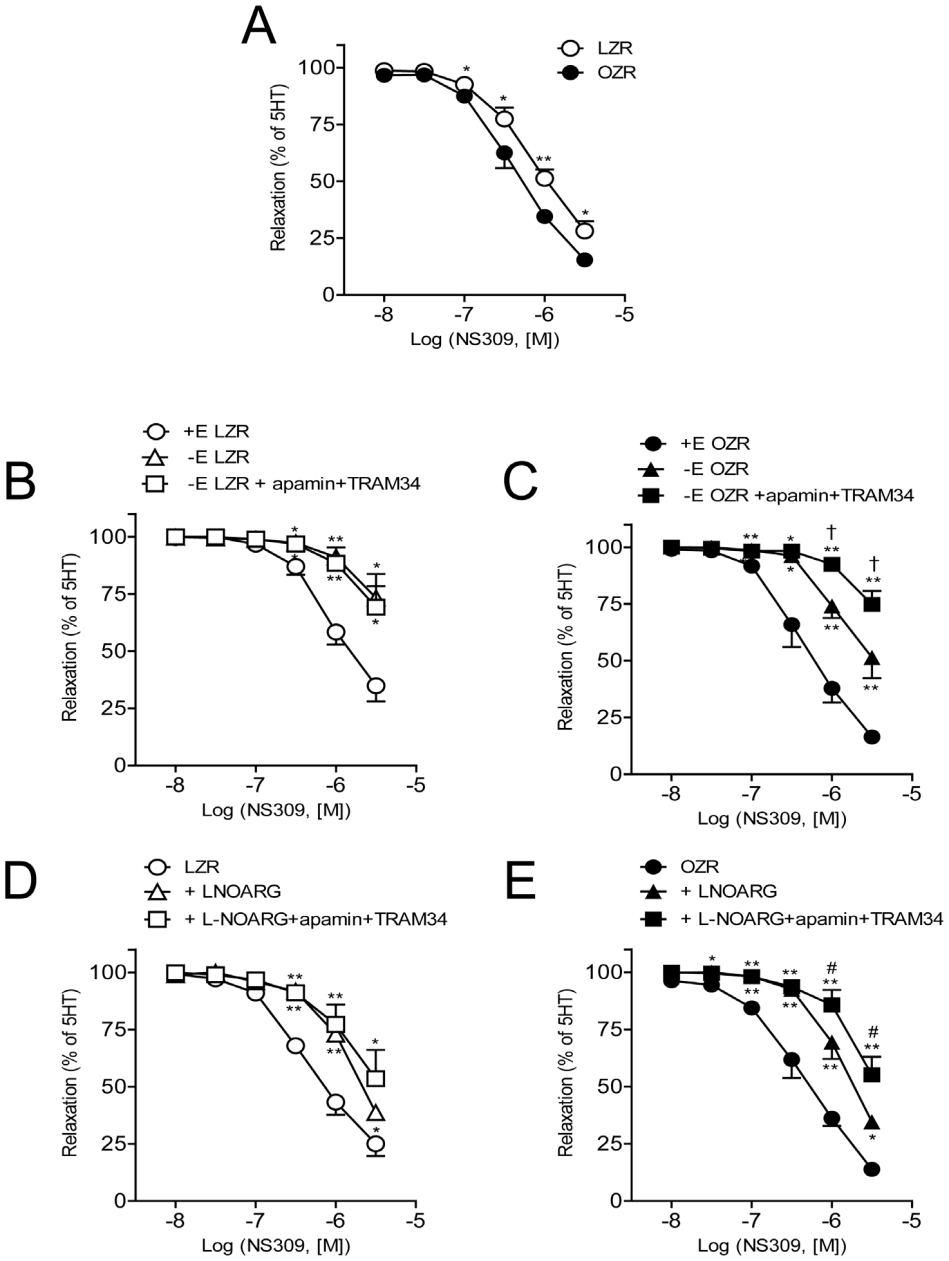
Endothelial SK3/IK1 channels activation contributes to NO-induced relaxation in coronary arteries from LZR and OZR. (A) The selective opener of SK3 and IK1 channels NS309 induces a larger relaxant effect on coronary arteries of OZR. (B, C) Effect of endothelium removal and further blockade of SK3/IK1 channels with apamin (0.5 µM) plus TRAM (0.4 µM) on the relaxations induced by NS309 in coronary arteries of LZR (B) and OZR (C). (D, E) Effect of NOS blockade with L-NOARG (100 µM) and further inhibition of SK3/IK1 channels with apamin plus TRAM on the relaxations elicited by NS309 in coronary arteries of LZR (D) and OZR (E). Results are expressed as a percentage of the contraction induced by serotonin. Points represent mean ± SEM of n = 9–13 arteries (1–2 arteries per animal). Significant differences from controls were analyzed using unpaired t-test *P<0.05; **P<0.01 *vs* control (Fig 3 A); or one way ANOVA followed by a Bonferroni test *P<0.05 and **P<0.01 *vs* control; ^†^ *P<*0.05 *vs* –E; *^#^P*<0.05 *vs* L-NOARG (Fig 3 B–E).

To further elucidate the role of SK3/IK1 channels in the coronary relaxations mediated by endothelial NO, arteries pre-contracted with 5-HT were stimulated with increasing concentrations of the selective opener of SK3/IK1 channels NS309. This drug elicited relaxations in arteries from both LZR and OZR, and both sensitivity and responses to the maximal concentration of NS309 were enhanced in arteries from OZR (Fig 3 A).

Mechanical removal of endothelium was performed to explore the contribution of endothelial SK3/IK1 channels to the NS309-induced relaxations. Endothelium removal blunted NS309-induced relaxations in arteries of LZR and to a lesser extent in OZR (Fig 3 B–C). Inhibition of SK3/IK1 channels with a combination of apamin plus TRAM34 did not further decrease NS309-induced relaxations in endothelium-denuded arteries from LZR whereas this procedure induced an additional blockade in arteries of OZR (Fig 3 B–C). Treatment with apamin plus TRAM34 increased basal tone in endothelium-denuded arteries of OZR by 24±5 of the KPSS-induced contraction (n = 6).

 Since relaxations to ACh in coronary arteries from OZR are mostly dependent on NO release [26], the contribution of SK3 and IK1 channel activation to endothelial NO-mediated coronary relaxations was assessed. Inhibition of NOS with L-NOARG largely reduced NS309-induced relaxations in both lean and obese animals (Fig. 3 D–E) whereas further inhibition of SK3/IK1 channels induced an additional modest inhibition only in OZR (Fig. 3 D–E). This suggests that the relaxation induced by activation of SK3/IK1 channels with NS309 is mostly mediated by NO in coronary arteries, although other non-NO related mechanism(s) may exist in obese animals.

### 2.4. Role of SK3 and IK1 channels in the ACh-induced changes in [Ca^2+^]_i_ in coronary endothelium from LZR and OZR

**Figure 4:**
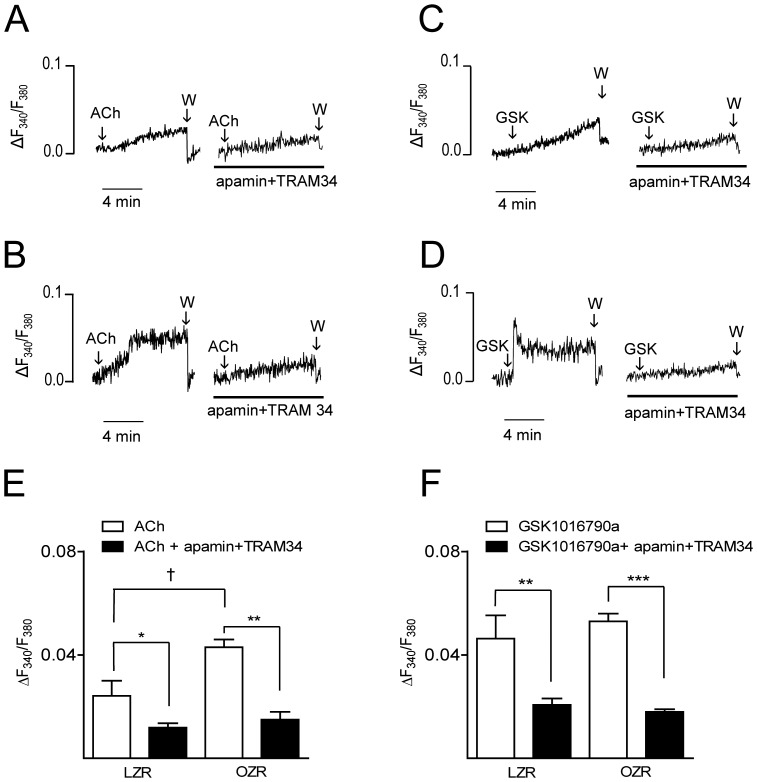
SK3 and IK1 channels are involved in the increased ACh-induced [Ca^2+^]_i_ mobilization in endothelial cells from OZR. Original recordings showing (A, B) the acetylcholine (ACh) (3 µM) and (C, D) GSK1016790a (1 µM)-induced increases in endothelial cell [Ca^2+^]_i_ in the presence and in the absence of apamin (0.5 µM) plus TRAM34 (0.4 µM) in intact coronary arteries from LZR (A, C) and OZR (B, D). (E, F) Summarized data showing the changes in endothelial cell [Ca^2+^]_i_ in response to ACh (E) and GSK1016790a (F) in the presence and in the absence of apamin plus TRAM34 in LZR and OZR. Results are expressed as absolute values and are means ± SEM of 5–6 arteries, (1 artery from 1 animal). Significant differences were analyzed using paired t-test *P<0.05, **P<0.01, ***P<0.001 or one way ANOVA followed by a Bonferroni test for across-group comparisons; ^†^ *P*<0.05 ACh OZR *vs* ACh LZR.

Measurements of endothelial [Ca^2+^]_i_ were performed in intact coronary arteries to elucidate whether ACh-induced Ca^2+^ signaling is changed in coronary endothelium under conditions of obesity. Basal F_340_/F_380_ ratios were not significantly different in the coronary endothelium from LZR (0.82±0.02, n = 5) and OZR (0.94±0.11, n = 6). Stimulation of endothelial cells with ACh (3 µM) induced an increase in [Ca^2+^]_i_ that was significantly higher in arteries from OZR compared to LZR (Fig. 4 A, B, E). Treatment with apamin plus TRAM34 reduced to the same level the increase in [Ca^2+^]_i_ induced by ACh suggesting that enhanced activity of SK3/IK1 channels in the coronary endothelium may contribute to the larger endothelial [Ca^2+^]_i_ mobilization in response to ACh in obese animals (Fig. 4 A, B, E).

The selective agonist of TRPV4 channels GSK1016790a [37] induced increases in endothelial cell [Ca^2+^]_i_ that were of similar magnitude in arteries from LZR and OZR, and also significantly reduced after treatment with apamin plus TRAM34 in both lean and obese animals (Fig. 4 C, D, F).

### 2.5. Immunohistochemical localization and expression of SK3 and IK1 channels in coronary arteries

**Figure 5:**
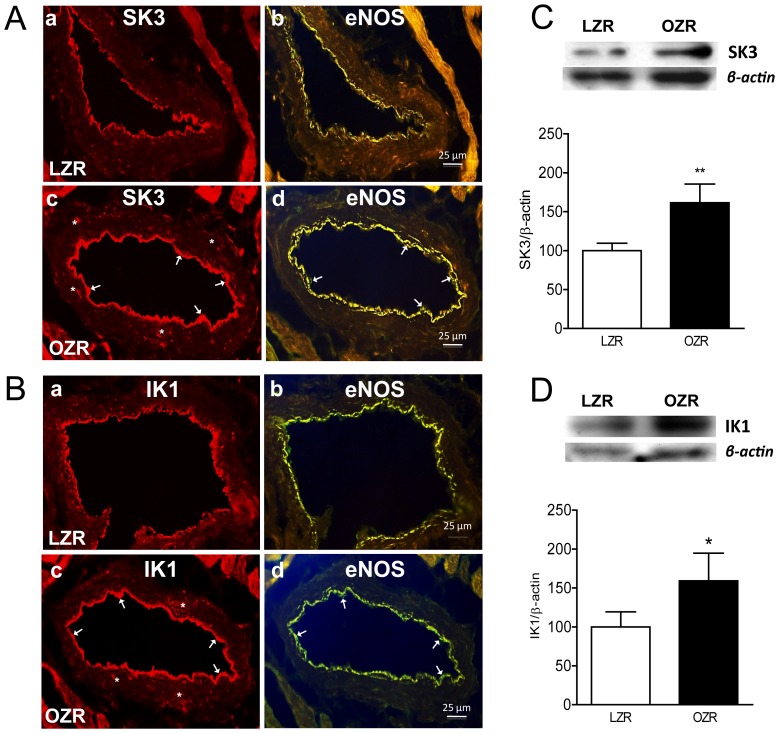
SK3 and IK1 channel expression in coronary arteries from LZR and OZR. (A, B) Immunohistochemical labeling of SK3 (A) and IK1 (B) channel in coronary arteries from LZR (top pannel) and OZR (bottom pannel). SK3 (Aa, Ac) and IK1 (Ba, Bc) channel immunoreactivity (arrows) was mainly located in the endothelium and eventually in the smooth muscle layer (asterisk) and was higher in arteries from OZR compared with LZR (Ac and Bc, respectively). Sections are representative of n = 3 OZR and n = 3 LZR, (1 artery per animal). (C, D) SK3 and IK1 channel expression is increased in coronary arteries from OZR. Western blot analysis of SK3 (C) and IK1 (D) channel expression in coronary arteries from LZR and OZR. Results were quantified by densitometry. Data are shown as means ± SEM of 5–7 animals. Significant differences from controls were analyzed using unpaired t-test *P<0.05; **P<0.01 *vs* LZR.

Figure 5 illustrates the expression and localization of SK3 (Fig 5 A–C) and IK1 (Fig 5B–D) channels in cross sections of coronary arteries from LZR and OZR. Immunoreactivity for SK3 and IK1 channels was found mostly in the endothelial layer of both LZR and OZR and was higher in arteries from obese animals. SK3 and IK1 channel immunoreactivity was occasionally found in small clusters distributed in the smooth muscle layer mainly in arteries from OZR. To confirm these findings using more quantitative methods, we next assessed SK3 and IK1 channel protein levels using western blot analysis, which were significantly increased in coronary arteries from OZR (Fig. 5 C and Fig. 5 D).

## Discussion

This study provides new findings coupling activation of SK3 and IK1 channels to endothelial cell Ca^2+^ and NO-mediated relaxations of coronary arteries in obesity. The contribution of these channels to the ACh relaxant responses is markedly increased in coronary arteries of obese animals. SK3/IK1 channels are involved in the ACh-induced [Ca^2+^]_i_ mobilization in the endothelial cell that is also increased in obese animals in part due to the enhanced activity of SK3/IK1 channels. Enhanced function is accompanied by upregulation of SK3/IK1 channels in coronary arteries in obesity.

The role of endothelial SK3 and IK1 channels in the EDH-mediated responses has generally been assumed as a parallel or compensatory pathway to NO-mediated endothelium-dependent dilation, suggesting the importance of these endothelial channels for the EDH-derived relaxation when NO and prostacyclin are compromised [1], [2], [4]. However, SK3/IK1 channels can modulate the availability of endothelium-derived NO, since their opening is associated with the activation of endothelial NOS [8]–[11]. The present study demonstrates that while the contribution of SK3/IK1 to the endothelium-dependent NO-mediated ACh relaxations was minor in coronary arteries from LZR, this contribution was significantly augmented in arteries from obese animals. Furthermore, ACh relaxant responses were confirmed to be largely dependent on NO release in both LZR and OZR [26], [32]. The fact that the relaxations induced by NS309 were blunted by endothelial removal and by NOS blockade in LZR further indicates that activation of endothelial SK3/IK1 channels might be involved in the endothelial NO-mediated relaxations of healthy coronary arteries. This is supported by recent studies showing that NS309 and the new selective opener of SK3 and IK1 channels SKA-31 markedly enhanced coronary blood flow [38], [39], these vasodilator responses being mediated by NO [38].

Obesity is associated with endothelial dysfunction and diminished NO bioavailability, mainly as a consequence of oxidative stress and the proinflammatory state of the vascular wall [15]–[17]. However, EDH-mediated responses seem to be preserved at least in the early stages of obesity due to maintained function of SK3/IK1 channels [18]–[21], [40]. In this regard, unchanged [19] or even increased [18] EDH-mediated relaxations evoked by ACh in peripheral arteries of obese mice have been reported. Moreover, vascular IK1 channel function and expression were found to be upregulated in obese rats [21] and endothelium-dependent relaxation was shown to undergo a shift from entirely NO-mediated mechanisms to IK1-dependent pathways that accounted for EDH activity in obese animals [20]. In contrast, a reduced EDH-mediated response was found in OZR due to decreased myoendothelial gap junction expression, but SK3/IK1 channel function remained likewise unaltered [41].

In the present study, we have also found a shift in the contribution of SK3 and IK1 channels to the endothelium-dependent relaxations of coronary arteries in obese animals. Thus, blockade of these channels produced a significantly greater inhibition of the ACh relaxant responses in OZR compared to healthy controls. Moreover, relaxations induced by NS309 were enhanced and Western blot analysis showed a marked increase in protein expression of both SK3 and IK1 channels in the coronary artery wall of obese animals. In contrast to that reported in obesity [21] and type 2 diabetes [42], augmented contribution to the endothelial vasodilator responses was not restricted to IK1 but also to SK3 channels, this preservation suggesting the functional relevance of the latter in the coronary circulation. Both SK3 and IK1 channels are involved in the non-chemical EDH-mediated response that is electrotonically transferred to the vascular myocytes in coronary arteries [43]. IK1 channels are usually found in the endothelial projections through the inner elastic lamina and SK3 channels are preferentially located within the endothelial caveolae and nearby homocellular gap junctions in the arterial wall [3], [44]. These different subcellular localizations might explain the diverging roles reported for SK3 and IK1 channels: the IK1 channel seems to be of pivotal importance in the EDH-mediated response, whereas SK3 channel deficiency preferably impairs ACh-induced NO-mediated relaxation [45]. Accordingly, upregulation of SK3 channels found in the coronary endothelium of obese animals would be consistent with the preserved NO-mediated relaxations reported in these arteries [26], [32].

The mechanism underlying increased expression of SK3 and IK3 channels in coronary arteries of obese rats remains to be elucidated. Recent studies have shown that laminar shear-stress increased expression of SK3 and IK1 channel mRNAs in cultured human coronary artery endothelial cells via the Akt signalling cascade [46]. Moreover, advanced glycation end products up-regulated IK1 channels in cardiac fibroblasts through a pathway also including PI3K/Akt signals [47]. Although OZR exhibit mild hyperglycemia, hyperinsulinemia in this strain has been found associated to up-regulation of the endothelial PI3K/Akt signalling pathway [32], which might account for the increased expression of SK3 and IK1 channels shown in the present study.

Whereas SK3 channels have earlier been reported in VSM of coronary arteries [48], is generally accepted that healthy contractile VSM cells do not express IK1 channels [49]. Up-regulation of the latter has been reported to be involved in VSM proliferation [49] and also to be related with the development of coronary atherosclerosis in vivo [50]. In this regard, functional experiments in the present study indicate an enhanced IK1 and to a lesser extent SK3 channel function involved in the relaxations induced by exogenous NO in coronary arteries of obese animals, which might additionally account for the preserved ACh-induced relaxation observed in OZR [25], [26]. In support of these findings, the immunohistochemical images revealed that despite expression of SK3/IK1 channels takes place mainly in the endothelial layer, immunoreaction for both channel proteins can be found scattered in the VSM layer in OZR. Moreover, enhanced endothelium-independent SK3/IK1 activity involved in the maintenance of basal tone was found in OZR. Relaxations elicited by activation of both channels with NS309, are mostly dependent on the endothelium and on the release of NO in healthy coronary arteries. Strikingly, these relaxations exhibited an endothelium-independent NOS inhibitor-resistant component that was blocked by apamin plus TRAM34 in obese animals. Hence, these findings suggest a role of VSM SK3/IK1 channels in the relaxations of coronary arteries in obese animals. The pronounced inhibitory effect of TRAM34 on the relaxant responses to the NO donor SNAP in OZR further suggests a role for VSM IK1 channels in the NO relaxant responses of coronary arteries from obese animals. Technical limitations in the present study, due to the restricted amount of protein obtained from the rat left descending coronary artery, prevented the assessment of the differential increase in SK3/IK1 channel expression in endothelium and VSM of coronary arteries from obese rats. The mechanism(s) by which NO may activate VSM SK3/IK1 channels to contribute to the NO-induced smooth muscle relaxation in OZR is unknown but the NO/cGMP signaling pathway might be involved, as in the case of VSM BK_Ca_ channels [51]. Accordingly, this pathway has recently been reported for the agonist of the alpha estrogen receptor propyl pyrazole triol that causes vascular relaxation via activation of VSM IK1 channels through the NO/cGMP signaling cascade [52].

Taken together, the present findings demonstrate an enhanced contribution of SK3 and IK1 channels to the endothelium-dependent relaxations of coronary arteries in insulin resistant obese rats, through both increased NO release and EDH-mediated responses. This is in agreement with the enhanced activity and contribution of VSM BK_Ca_ channels to the maintained EDH-mediated responses of coronary arterioles recently reported in a rat model of high-fat diet-induced obesity [31], which suggests that up-regulation of K_Ca_ channels is a mechanism to preserve coronary vasodilatation in obesity.

Stimulation of cultured endothelial cells with ACh has been shown to be associated with an initial transient rise in [Ca^2+^]_i_ due to inositol 1,4,5-trisphosphate-mediated Ca^2+^ released from intracellular stores followed by a sustained elevation due to Ca^2+^ influx via TRP channels [53]. Rise in [Ca^2+^]_i_ in endothelial cells is a key signaling event that triggers the synthesis and release of endothelium-derived relaxing factors including NO and prostacyclin and activates the EDH-mediated response [2]. In the present study, ACh induced an increase in endothelial [Ca^2+^]_i_ of coronary arteries that was markedly enhanced in arteries from OZR. Increased agonist-induced [Ca^2+^]_i_ mobilization has been reported in cultured endothelial cells in response to hyperglycemia [54], although impaired rises in endothelial [Ca^2+^]_i_ coupled to reduced expression and activity of SK3 were recently reported in arteries of type 1 diabetic rats [55].

Upon stimulation with endothelial agonists, endothelial SK3 and IK1 channels are activated by local Ca^2+^ events from the inositol 1,4,5-trisphosphate receptors [56] and by Ca^2+^ signals due to Ca^2+^ influx through TRPV4 channels [37], [57]. Levels of endothelial cell [Ca^2+^]_i_ have been reported to be both independent [8], [58]–[60] or dependent on changes in membrane potential [5]–[7], [61]. In support of the former, endothelial [Ca^2+^]_i_ levels were shown to be unaltered by changes in membrane potential in response to activators of SK3/IK1 channels in intact segments of retinal arteries [60]. Furthermore, a combination of SK3/IK1 channels inhibitors neither affected agonist-induced increases in endothelial [Ca^2+^]_i_ in intact rat mesenteric arteries [8], [58], [59]. The idea that activation of both SK3 and IK1 channels is coupled to NO release is based on the assumption that SK3/IK1 channels-induced hyperpolarization in endothelial cells might increase the driving force for the influx of Ca^2+^ via TRP channels thereby sustaining the activating Ca^2+^ signal [5]–[7], [61]. Thus, inhibition of SK3/IK1 channels was associated with a decreased agonist-induced [Ca^2+^]_i_ rise and NO release in cultured endothelial cells [9]. In addition, selective activation of these channels with NS309 increased [Ca^2+^]_i_ and NO formation in human umbilical vein endothelial cells and freshly isolated endothelial cells [10], [62]. Moreover, recent reports of intact arteries have confirmed the involvement of endothelial IK1 and to some extent SK3 channels in the positive feedback mechanism that influences endothelial Ca^2+^ dynamics upon agonist stimulation [7]. In addition, both pharmacological blockade and SK3/IK1 deficiency reduced the frequency of ACh-stimulated Ca^2+^ dynamics in mice mesenteric arteries [7]. Accordingly, in the present study a combination of apamin plus TRAM34 reduced the profile of rises in endothelial [Ca^2+^]_i_ in response to ACh stimulation in coronary arteries. Hence, this suggests that activation of SK3/IK1 channels in the present study is involved in the ACh-induced mobilization of endothelial [Ca^2+^]_i_ and it is consistent with the idea that Ca^2+^ influx in endothelial cells is driven, to a significant extent, by SK3/IK1 channel-induced membrane hyperpolarization.

On the other hand, SK3 and IK1 channels might be in part responsible for the higher ACh-induced endothelial [Ca^2+^]_i_ mobilization observed in coronary arteries from obese animals, since treatment with apamin plus TRAM34 decreased endothelial [Ca^2+^]_i_ to a similar level in OZR and LZR arteries. This larger inhibition along with the increased expression of SK3 and IK1 channels in the coronary vasculature of obese animals suggest that activation of these channels might induce a larger endothelium hyperpolarization, therefore increasing the electrochemical driving force for Ca^2+^ entry into endothelial cells. This in turn would explain the higher rise in endothelial [Ca^2+^]_i_ upon ACh stimulation in arteries from obese rats. Among all TRP channels, TRPV4 is ubiquitously expressed in endothelial cells in close proximity to K_Ca_ channels and has been involved in agonist-induced Ca^2+^ influx and NO production [37] in endothelial cells leading to relaxation [7], [57], [63]. In the present study, stimulation of the quiescent coronary endothelium with a specific agonist of TRPV4 channels induced a rise in endothelial [Ca^2+^]_i_ of similar magnitude in arteries from both lean and obese rats that was blunted by blockade of SK3/IK1 channels. This is in agreement with recent studies demonstrating that endothelial agonist-induced Ca^2+^ influx through TRPV4 channels leads to IK1 channel activation and endothelial cell hyperpolarization [57]. Thus, TRPV4-mediated Ca^2+^ influx and IK1-hyperpolarization are essential to the Ca^2+^ amplifier feedback mechanism that sustains the driving force for Ca^2+^ influx into endothelial cells [7]. Whether Ca^2+^ entry through TRPV4 channels contributes to the enhanced endothelial [Ca^2+^] mobilization in response to ACh observed in coronary arteries from obese rats requires further elucidation.

In summary, the observed upregulation of SK3 and IK1 channels might contribute for the signaling pathway that compensates and preserves coronary relaxation by increasing endothelial [Ca^2+^]_i_ in early stages of obesity. Whether upregulation of SK3/IK1 channels continues to be beneficial in the long term, or by contrast it might contribute to endothelial damage needs to be answered. In this regard, a limited spread of relaxation mediated by increased activation of SK3/IK1 channels in aging has recently been reported [64]. Moreover, up-regulation/induction of IK1 channels has been involved in VSM proliferation[49] and also has been related with the development of coronary atherosclerosis in vivo[50].

## Supporting Information

Table S1 **Supplementary material.** Metabolic parameters of LZR and OZR.
(DOC)
