# Supplementary material for: Upregulation of SK3 and IK1 Channels Contributes to the Enhanced Endothelial Calcium Signaling and the Preserved Coronary Relaxation in Obese Zucker Rats
Source: PLoS One. 2014 Oct 10;9(10):e109432. doi: 10.1371/journal.pone.0109432 (PMC4193814; doi:10.1371/journal.pone.0109432)
Supplement: Table S1 — Supplementary material. Metabolic parameters of LZR and OZR. (DOC) [file pone.0109432.s001.doc]

**Table S1.** Metabolic parameters of LZR and OZR.

|  | ***LZR*** | ***n*** | ***OZR*** | ***n*** |
| --- | --- | --- | --- | --- |
| Body weight, g | 363 ± 10 | 36 | 479 ± 7 | 37 |
| Blood glucose concentration, mg/dl | 106 ± 9 | 10 | 164 ± 21* | 12 |
| Plasma insulin concentration, ng/ml | 1,1 ± 0,1 | 10 | 3,6 ± 0,2‡ | 12 |
| Plasma total cholesterol concentration, mg/dl | 82 ± 8 | 10 | 313 ± 38‡ | 6 |
| Plasma triglyceride concentration, mg/dl | 25 ± 3 | 6 | 79 ± 5‡ | 6 |

Data are means ± SE; n, number of animals. Lean Zucker rats (LZR) and Obese Zucker Rats (OZR) of 17–18 wk of age were studied. Significant differences were analyzed by an unpaired Student’s t-test. *P< 0.05 and ‡P< 0.0001 *vs* LZR.
